# The motor pattern of rolling escape locomotion in *Drosophila* larvae

**DOI:** 10.1242/jeb.252063

**Published:** 2026-09-04

**Authors:** Liping He, Lydia J. Borjon, W. Daniel Tracey Jr

**Affiliations:** Gill Institute for Neuroscience, Department of Biology, Indiana University, Bloomington, IN 47405-2204, USA

**Keywords:** Nociception, Escape behavior, Sensorimotor, Muscle, Motor neurons, Premotor, Neurons

## Abstract

When undisturbed, *Drosophila* larvae move forward through their environment with sweeping waves of caudal to rostral muscle contractions. In stark contrast, nociceptive sensory stimuli trigger the larvae to roll across the substrate by corkscrewing around the long body axis. Here, we have determined the detailed timing of muscle activation that generates the body rotation across abdominal segments. To do so, we developed a high-speed confocal time-lapse imaging preparation that allowed us to trigger rolling with nociceptor optogenetics while simultaneously imaging a genetically encoded calcium sensor expressed in the muscles. Of the 30 muscles present in each larval abdominal hemisegment, we found that only 11 muscles are consistently and specifically activated across segments during rolling. Eight additional muscles are more sparsely activated. Importantly, the sequential pattern of muscle recruitment during rolling is completely distinct from that of forward or reverse crawling. A roll involves a wave of muscle activation that propagates around the larval circumference (in the transverse plane of each segment) and involves four co-active muscle groups. A pattern of activation progresses from co-active ventral muscle groups to dorsal groups and then spreads across the midline to the contralateral dorsal muscle groups, then progresses back to the ventral groups. The direction of a roll is determined by the clockwise or counterclockwise order of muscle group activation around the transverse plane. Finally, an analysis of the connectivity of inputs to larval motor neurons revealed a ring-like network architecture that could support the sequence of activation of the rolling motor pattern.

## Teaser

**Summary:** Rolling escape behavior in *Drosophila* larvae involves muscle recruitment completely distinct from that of forward/reverse crawling, with direction determined by the order of muscle group activation, supported by a ring-like motor neuron network.

## INTRODUCTION

Animal locomotion is generated by coordinated patterns of neural activity and muscle contraction. Depending on the neural architecture and body plan, this can result in a variety of movement patterns. *Drosophila* larvae, which have a simple body plan and a small nervous system, are able to perform multiple modes of locomotion. Like many other vermiform organisms, fly larvae primarily move around by crawling, forwards or backwards, using peristaltic waves of muscle contractions along the longitudinal axis of their body (Heckscher et al., 2012; Zarin et al., 2019). In addition, fly larvae have a specialized form of locomotion called rolling, which is a vital defense behavior against natural threats such as attacks by parasitoid wasps that attempt to infect larvae with their eggs. To escape, *Drosophila* larvae rotate their body in a corkscrew-like fashion to dislodge the attacking wasp (Hwang et al., 2007; Robertson et al., 2013). Unlike bilaterally symmetric movements such as crawling, rolling is asymmetric, with body bending and rotation propagating around the circumference of the larval body. Rolling is typically initiated by a unilateral muscle contraction that creates a characteristic C-bend, which is followed by rapid body rotations (Tracey et al., 2003). We sought to understand the pattern of muscle activation that generates the rotation of the escape rolling behavior.

The larval body is bilaterally symmetric and contains three thoracic (T1–T3) and nine abdominal segments (A1–A9), where the last two abdominal segments form specialized structures at the posterior end. Segments A1–A7 contain 30 pairs of segmentally homologous muscles that are layered below the epithelium, with the exception of muscle 25, which is not present in segment A1. The body wall muscles are arranged longitudinally, obliquely and orthogonally relative to the larval body axis, and can be identified by shape, orientation and position. The muscles are innervated by segmental and intersegmental nerves, which contain the efferent motor neurons. The majority of motor neurons connect to only one muscle in a one-to-one mapping, while a subset of motor neurons have broad connections to spatially organized muscle groups. Upstream premotor interneurons project to multiple motor neuron targets, and each motor neuron receives inputs from multiple premotor neurons from the ipsilateral and contralateral side (Zarin et al., 2019). However, the premotor neuron circuits that control muscle activity during rolling are not understood.

Components of the sensorimotor circuit that evokes rolling have been identified in previous studies, but the complete network of neurons that controls this behavior is not known. Rolling can be triggered not only by wasp attacks but also by stimulation with noxious heat, chemicals and mechanical stimuli. This sensory information is detected by multimodal high-threshold sensory neurons called class IV dendrite arborization (cIVda) neurons, which innervate the entire larval epidermis with large receptive fields (Grueber et al., 2002; Hwang et al., 2007; Tracey et al., 2003). The sensory information is transmitted to multiple types of interneurons in the ventral nerve cord, including Basins, DnB, DP-ilp7, A08n and mCSIs, which integrate information from multiple sensory modalities, project onto higher order interneurons (Burgos et al., 2018; Hu et al., 2017; Ohyama et al., 2015; Yoshino et al., 2017; reviewed in Chin and Tracey, 2017), and ultimately connect to a motor program that carries out the behavior. Optogenetic activation of neurons at several points in the circuit can evoke complete rolls. For example, a pair of command-like interneurons called Goro is sufficient to trigger rolling behavior (Ohyama et al., 2015). Rolling can also be evoked by activating second-order interneurons, such as Basins which connect to the Goro pathway (Ohyama et al., 2015), and mCSIs which trigger rolling independently of Goro (Yoshino et al., 2017), showing that there are multiple neural pathways that control this behavior. Optogenetic activation of cIVda sensory neurons is also sufficient to evoke rolls (Hwang et al., 2007), presumably by activating all components of the sensorimotor circuit as would be the case with a natural noxious stimulus.

In this study, we investigated the pattern of muscle activation that generates the body rotation of the rolling escape behavior using high-speed confocal time-lapse imaging combined with optogenetic cIVda activation. Our approach contrasts with prior studies that investigated the motor output through optogenetic activation of the downstream Goro neurons (which, as noted above, are required for some but not all rolling pathways). We observed that a subset of segmentally homologous muscles becomes active in a circumferentially traveling wave with peak activation in the inner curvature of the C-bend of the rolling larva. This detailed analysis of the timing of activation of muscles across the abdominal segments provides important information for building a neural connectivity map that underlies the rolling motor program.

## MATERIALS AND METHODS

### *Drosophila* maintenance

Flies were maintained on standard Bloomington *Drosophila* Stock Center cornmeal-agar food at 22°C in a relative humidity of 70%. The strains used in this study were: *R44H10-LexA LexAop-GCaMP6f/CyO* (Zarin et al., 2019) and *ppk-GAL4 UAS-ChR2/CyO RFP(mCherry)*.

### Rolling behavior imaging

Optogenetic activation with channelrhodopsin2-YFP in *Drosophila* larvae requires dietary supplementation with *all-transretinal* (ATR). We found that rolling of early second instar larvae could be facilitated by prefeeding the ATR to their virgin mothers (with yeast paste containing 1 mmol l^−1^ ATR) for 3–4 days (presumably by maternal loading of ATR into eggs). We crossed the male *R44H10-LexA LexAop-GCaMP6f/CyO* flies to ATR fed female *ppk-GAL4 UAS-Chr2/CyO RFP*. After 3 days of mating in vials containing ATR-supplemented yeast paste, the flies were then transferred to apple juice plate cages with ATR yeast paste and allowed to lay eggs overnight in the dark in a 25°C incubator (70% humidity). Larvae were raised on the apple juice and ATR yeast paste until second instar (as scored by size of larvae).

For imaging, 3% agarose pads were cut into 10 mm L×10 mm W squares and a rounded hole in the center of the agarose pad was scooped out using a pipette tip. A small droplet of water was then used to fill the hole in the agar pad and a second instar larva of genotype *R44H10-LexA LexAop-GCaMP6f/ppk-GAL4 UAS-Chr2:YFP* was placed in the water-filled depression, and then covered with a 1.5 mm coverslip. The preparation was then placed coverslip down on an inverted microscope (Zeiss LSM V Live). Imaging was conducted with excitation by a 100 mW 488 nm laser at 50–80% power. This laser excitation was effective at triggering rolling (in a small fraction of tested larvae) due to expression of CHR2 in nociceptors. Time series images were acquired with a 10×/0.3 objective, a wide-open pinhole and a long-pass 505 emission filter at 60 frames s^−1^ for approximately 10 s. The thickness of an optical section in these imaging conditions was 119.5 µm and the larval diameter at this stage of development is approximately 250 µm. Our imaging conditions therefore allowed us to observe muscle activity in half the circumference of the larva at any given moment.

### Abdominal body wall muscle activity assay

Muscle activity was determined by the visual inspection of GCaMP6f signals from the time series recordings. Muscles were scored as ON based on a clearly observable rise in GCaMP6f pseudo-colored fluorescence and clearly visible shortening of the visible muscles. The 8-bit grayscale images were processed with a rainbow pseudocolor scale in which colors from blue to red indicate the intensity of GCaMP6f fluorescence. Inactive muscles were not scored as ON if their intensity resembled baseline GCaMP6f fluorescence. Our imaging settings allowed us to analyze individual muscle activity in half of the larvae in which muscles were visible and nearest to the coverslip.

Visual inspection of muscle activity was confirmed by measuring a subset of muscles using region of interest (ROI) analysis. ROI were selected manually frame-by-frame and the GCaMP6f fluorescence in ROI of muscles DL9, DL4 and VA26 from segment A4 was measured during larval rolling locomotion. The change of GCaMP6f fluorescence intensity in ROI (Δ*F*/*F*) was calculated and plotted versus the normalized time (NTime).

### Data analysis

The duration of a rolling cycle (ΔTime) was calculated as the time interval between two recording time points (at the start and end of a roll) when the larval ventral midline in segment A4 was centered in the image plane. The time stamp at the beginning of the rolling cycle (when segment A4 was centered on the ventral midline) was defined as TimeV. This allowed us to transform the true recording time to normalized time (NTime) from 0 to 1 as follows: NTime=(TimeR−TimeV)/ΔTime, where TimeR is a single time stamp from a frame in the recording and TimeV is as defined above. At NTime=0 the ventral midline is centered, at NTime=0.5 the dorsal midline of the larva is centered, at NTime=1 the ventral midline is again centered after the completion of a 360 deg roll (Fig. S1).

The 11 muscles consistently involved in rolling cluster into four groups based on the pairwise distances between pairs of activation curves (see below). To calculate the distance between two curves, we first calculated Euclidean distances between a point on curve 1 and all the points on curve 2 (time points from one to three segments are used in this paper). The shortest distance was used as the distance from a given point on curve 1 to curve 2. Then, we calculated the distance from all the other points on curve 1 to curve 2. Finally, we took the average of the distances from point to curve as the distance from curve 1 to curve 2. We calculated the pairwise distances of 11 muscle activation curves and cladograms were constructed from the pairwise distances using the ‘Nearest distance’ method.

### Connectivity network

The motor neuron graph networks were based on the connectivity matrix between premotor neurons (PMNs) and motor neurons (MNs) published by Zarin et al. (2019). The connectivity matrix provides directional weighted connections from PMNs to PMNs, and PMNs to MNs, where the connection weights are a percentage of the total synaptic inputs to each MN. From the bipartite network of PMN-to-MN connections (ignoring PMN-to-PMN connections), we generated a one-mode projection for the MN nodes using the formula proj_B=bip′ * bip where bip is the bipartite PMN×MN network, bip′ is its inverse, * is matrix multiplication and proj_B is the resulting MN×MN projection. The network was plotted using MATLAB R2024b (MathWorks) imagesc() function with colormap ‘hot’ to visualize the connectivity matrix (Fig. 6A), and graph() object and plot() function to visualize the network graph, with automatic node placement using the ‘force’ layout, which spaces distantly connected nodes further apart (Fig. 6B,C). For visual clarity, the graph networks were plotted with a threshold cutoff for edge weights >0.015 (Fig. 6B) or >0.005 (Fig. 6C). Communities were detected using the MATLAB functions from the Brain Connectivity Toolbox: community_louvain() repeated 100 times, from which a consensus community partition was determined using agreement() and consensus_und().

MATLAB was used to construct a permutation test to determine whether the sums of synaptic weights for the rolling network are statistically different from chance. For each connection type (excitatory, inhibitory or unknown×ipsilateral, or contralateral), random subsamples were taken from the whole network, equal in number to that connection type in the rolling network, using datasample(), 1000 times with replacement. The distributions of the sums of the permuted subsamples are shown as violin plots in Fig. 6E. To test whether the observed values from the rolling network lie significantly outside the permuted data, a two-tailed non-parametric test was used, in which the *P*-values were computed as twice the proportion of permuted null statistics falling at or beyond the observed values.

## RESULTS

**Fig. 1.**
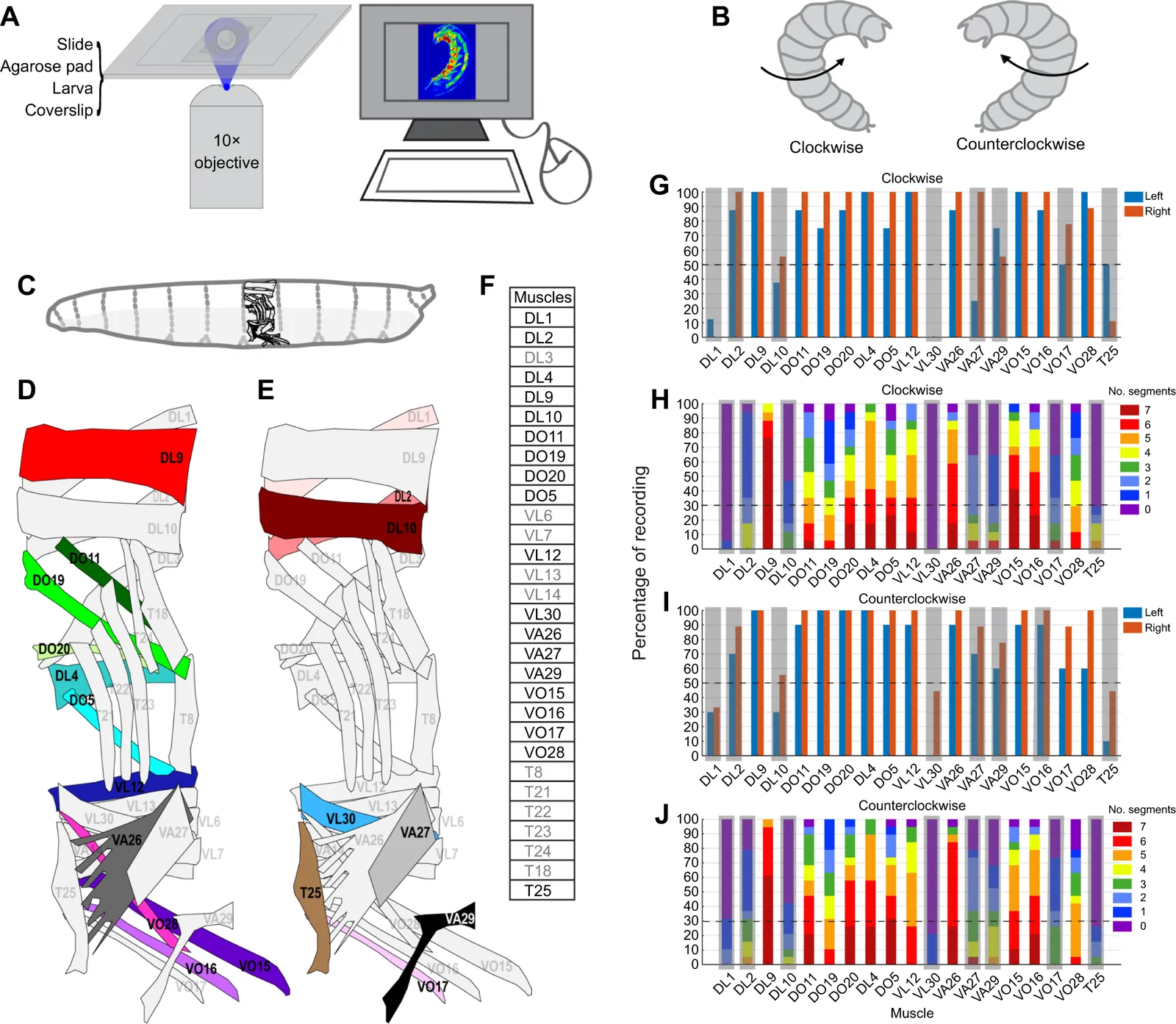
**The abdominal muscles that are activated during larval rolling behavior.** (A) Schematic drawing of the imaging preparation. The *Drosophila* larva was imaged on an inverted confocal microscope through a coverslip. An agar pad above the larva contained a water-filled depression and was mounted on a glass slide. (B) Schematic drawing of clockwise and counterclockwise larval rolling: heads of the larvae are up, tails are down towards the bottom of the image. (C) Schematic drawing of a *Drosophila* larva as viewed from the side; 30 body wall muscles are shown in the 5th abdominal hemisegment. (D) Muscles that were consistently activated during larval rolling, indicated by solid colors. (E) Muscles that were sparsely or rarely activated during larval rolling, indicated by solid colors. (F) List of the 30 abdominal muscles with the activated muscles shown in black. (G) The frequency of recordings where activation was observed is graphed for each of the muscles involved in clockwise rolling. (H) The number of segments (ranging from 0 to 7) that showed activation for each muscle across recordings for clockwise rolling. (I) The frequency of recordings where activation was observed is graphed for each of the muscles involved in counterclockwise rolling. (J) The number of segments (ranging from 0 to 7) that showed activation for each muscle across recordings for counterclockwise rolling. Gray shading in G–J indicates sparsely or rarely activated muscles.

In order to observe the activation pattern of muscles during larval rolling behavior, we used calcium imaging as an indicator of muscle activity. Second instar larvae were placed on a coverslip and imaged on an inverted confocal microscope (Fig. 1A). An agar pad with a water-filled cutout depression was placed on top of the coverslip to ensure that the larva remained in the field of view during the rolling motion. The larvae expressed the calcium indicator GCaMP6.0f (Chen et al., 2013) under the control of the muscle-specific driver *R44H10-LexA* (Zarin et al., 2019), and also expressed photoactivatable Channelrhodopsin2::YFP under the control of the cIVda-specific driver *ppk1.9-Gal4* (Hwang et al., 2007). Thus, the blue 488 nm laser allowed for simultaneous excitation of GCaMP in muscles, and optogenetic activation of cIVda sensory neurons, which triggers rolling.

Larvae can perform rolls towards the right side (clockwise) or towards the left side of their body (counterclockwise) (Fig. 1B). With optogenetic activation, the direction of the roll appeared to occur at random, and we obtained 19 time-lapse recordings with completed rolls, of which 9 were clockwise and 10 were counterclockwise. The duration of a rolling cycle (i.e. the amount of time required for the larvae to complete a full 360 deg rotation) was 1.60±0.36 s (mean±s.d., *n*=9) for clockwise rolling and 1.70±0.46 s (mean±s.d., *n*=10) for counterclockwise rolling. As the larval body rotates, muscles disappear into the inner curve of the C-bend and then reappear on the other side. Through the microscope objectives, it was possible to observe either the side of the larva on which the muscles contract into the inner curve of the C-bend, or the side on which they relax and emerge from the inner curve. Regardless of the perspective of the viewer, the roll is driven by the same motor program. We chose to focus on rolling events that allowed us to observe and characterize the pattern of muscle activation as the muscles contract to drive body rotation.

Using careful frame-by-frame analysis, we painstakingly scored the activation of individual muscles in abdominal segments 1–7 (A1–A7) during rolling. A well-trained investigator can identify each of the 30 muscles in each larval hemisegment according to morphology, orientation, position on the dorsal–ventral axis and position in relation to other nearby muscles. The recordings were pseudocolored to represent fluorescence intensity, making it possible to score obvious increases in fluorescence. This resulted in a qualitative/binary ON or OFF designation of muscle activity. Although our imaging conditions did not allow for comprehensive quantitative measures of fluorescence in all muscles, the large magnitude of change of GCaMP6.0f intensity made the ON determination clear and unambiguous (see Movies 1 and 2). A caveat of this methodology is that small changes in the calcium indicator could not be reliably studied, and thus we cannot eliminate the possibility that we missed very weakly activated muscles in our analysis. To validate our visual scoring of muscle activity, we tracked the fluorescence of three pairs of muscles – DL9, DL4 and VA26 – in segment A4 across multiple roll events using ROI analysis. These quantitative results support our qualitative determination of muscle activity (see below).

### A subset of muscles is involved in rolling

We found that 11 of the 30 muscles in each abdominal hemisegment were consistently and robustly activated across recordings in both clockwise and counterclockwise rolling behavior: dorsal longitudinal muscles (DL) 9 and 4, the dorsal oblique muscles (DO) 11, 19, 20 and 5, the ventral longitudinal muscle (VL) 12, the ventral acute muscle (VA) 26 and the ventral oblique muscles (VO) 15, 16 and 28 (Fig. 1C–J). We observed that these muscles were each activated in three or more body segments in over 70% of recordings. Two other muscles (DL2 and VA29) were also activated in greater than 70% of recordings but in a sparser pattern that was rarely seen in more than three segments (Fig. 1G–J). An additional six muscles were more rarely activated (DL1, DL10, VL30, VA27, VO17 and T25) (Fig. 1E–J). Note that because muscles VA27 and VO17 are in close proximity to other strongly activated muscles, we could not unambiguously score their activity, so designating them as rarely activated muscles may be an underestimate. For the remaining 11 muscles, no activity was observed. Interestingly, although we made a focused effort, we did not observe activation of the transverse muscles T21, T22 or T23, which have previously been proposed to be important for rolling behavior (Yoshino et al., 2017). We verified that the R44H10-LexA driver is robustly expressed in the transverse muscles (data not shown; Zarin et al., 2019); however, we cannot exclude the possibility that these muscles are weakly activated during rolling, or that they are activated at a phase of the rolling cycle that was difficult to observe (i.e. in the C-bend, see below).

### A sequence of muscle contractions around the transverse circumference occurs during clockwise rolling

We next analyzed the sequential timing of the activation of the muscles involved in rolling. In the majority of our recordings, the C-bend began with body flexion on the ventral side. However, the exact initiation site of the C-bend, as well as the duration of each complete roll, varied between recordings. In order to compare the muscle activation pattern that propagates the body rotation in different rolling events, we identified anatomical landmarks from which to determine a normalized time scale (Fig. S1). We defined the beginning of a rolling cycle (normalized time, NTime 0) to be the point during a roll when the ventral midline of segment A4 was precisely centered relative to the left–right axis. The midpoint of a rolling cycle (NTime 0.5) was defined as the point when the dorsal midline of segment A4 was precisely centered. The end of the cycle (NTime 1) was when the ventral midline was again centered after a complete rotation. Note that the rolls actually initiate slightly prior to time 0 of the rolling cycle.

We carefully observed frame-by-frame still images (∼60 Hz) from our recordings and noted the time stamp at which each muscle first became activated in every abdominal segment A1–A7. We also noted the duration for which each active muscle remained clearly visible and identifiable in the recording. It was not possible to keep track of individual muscles for the entire rolling cycle for two reasons. First, our imaging setup allowed us to view the larvae only from one angle, and the depth of field under our imaging conditions encompassed only approximately half of the larva (in the sagittal/longitudinal plane) at any given time. Second, as strongly activated muscles turned into the C-bend, their bright fluorescence blended together and individual muscles could no longer be resolved. Therefore, although we were able to precisely determine the start time of muscle activation, we were not able to observe or accurately score when muscle activation ended.

**Fig. 2.**
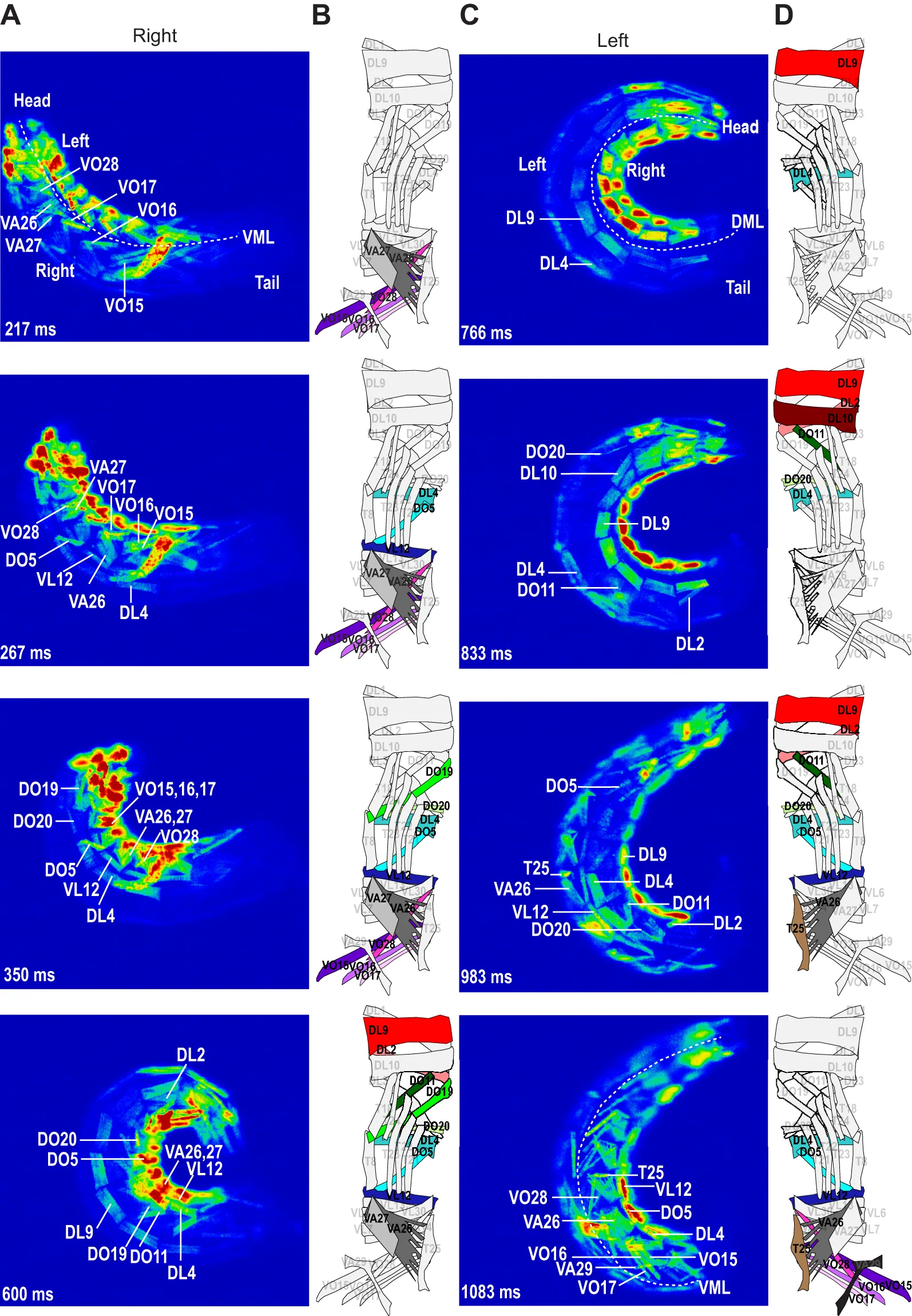
**The sequentially recruited body wall muscles during clockwise rolling.** (A,C) Selected frames from a recording of muscle activity of right (A) and left (C) hemisegments during clockwise rolling behavior. Colors from blue to red represent the intensity of the GCaMP6f signal from low to high (see Movie 1). Dashed lines indicate the ventral (VML) and dorsal (DML) midline. A subset of visibly activated muscles is labeled. ‘Right’ and ‘Left’ denote the corresponding sides of the larval body. (B,D) Schematic representation of the visibly activated muscles in larval abdominal right (B) and left (D) hemisegments for the frames shown in A and C. Solid colors indicate the initially activated muscles that are visible in the frame to the left.

**Fig. 3.**
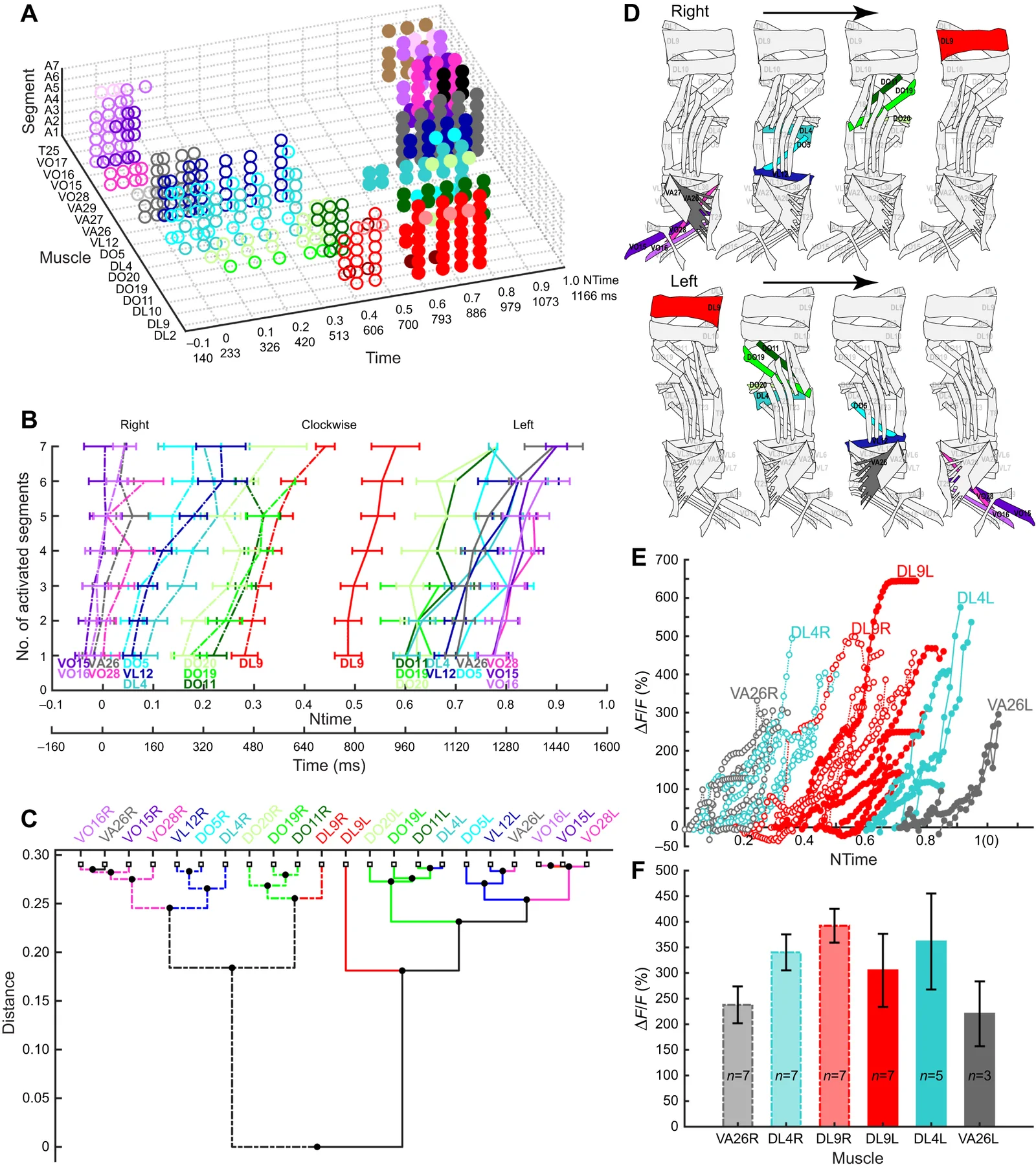
**The sequence of muscle recruitment during clockwise rolling.** (A) A 3D scatter plot depicting the sequence of activated muscles across the abdominal segments during clockwise rolling, from the right side (open circles) to the left side (filled circles). The *z-*axis shows the seven segments (A1–A7), the *y*-axis indicates the identity of individual muscles and the *x*-axis shows the time normalized to the rolling cycle (NTime) as well as the true time from the recording (see Movie 3 for the recording of this rolling event). Time in ms was obtained by multiplying NTime by the average duration of a rolling cycle. (B) Plot of the average recruitment timing for the 11 abdominal body wall muscles that are consistently activated during larval clockwise rolling. Error bars represent s.e.m. (*n*=9). The *x*-axis labels show the normalized rolling phase and the average minimum–maximum normalized time, respectively. (C) Cladogram depicting the relationship of recruitment timing of 11 commonly activated muscles in clockwise rolling. Four co-activated groups of muscles are color coded. Solid branch lines denote the left side of the larva and dashed lines denote the right side of the larva. (D) Schematic drawing showing the sequential activation of co-active muscle groups, proceeding from the right to the left side of the larval body during a clockwise roll. (E) Time series of GCaMP6f change in fluorescence (Δ*F*/*F*) in muscles VA26, DL4 and DL9 of abdominal segment A4 from seven recordings during clockwise rolling behavior, until the muscles disappear into the innermost curve of the C-bend. Solid lines and solid circles indicate the left hemisegment (L); dashed lines and open circles indicate the right hemisegment (R). (F) Average of the highest measurable Δ*F*/*F* across the recordings in muscles DL9, DL4 and VA26 of abdominal segment A4 during larval clockwise rolling. Error bars indicate the s.e.m.

The muscles that are involved in rolling are recruited in a sequential pattern around the circumference of the larval abdominal segments. In a typical clockwise rolling cycle, a prominent ventral flexion was observed near NTime 0, primarily involving muscles VO15R, VO16R, VO28R and VA26R (Figs 2 and 3A). This contraction then spread unidirectionally toward the right dorsal side of the body with recruitment of muscles VL12R, DO5R and DL4R and then spreading to DO20R, DO19R and DO11R, and finally to muscle DL9R (Figs 2 and 3A). The pattern of muscle contraction then crossed the dorsal midline, near NTime 0.5, to recruit muscles from the left side of the body in reverse order compared with the right side, beginning with the left dorsal muscles and spreading towards the left ventral side: DL9L was followed by DO11L, DO19L, DO20L, then DL4L, VL12L, VA26L and finally VO28L, VO15L and VO16L to complete the rolling cycle at NTime 1 (Figs 2 and 3A). The example rolling event shown in Figs 2 and 3A also includes activity in a few muscles that were less commonly observed in our recordings.

In stark contrast to peristaltic locomotion, homologous muscles are recruited nearly simultaneously across abdominal segments during rolling. This suggests that the neural circuitry underlying rolling is organized primarily around muscle identity, coordinating the activation of corresponding muscles across segments, rather than around the segmental recruitment logic that governs crawling. Fig. 3A summarizes the timing of muscle activation across all seven abdominal segments, showing that most homologous muscles became active in the majority of segments within a short amount of time. Thus, for the muscles that are involved in rolling locomotion, the homologous muscles activate across the whole anterior–posterior axis, and this activation propagates circumferentially from the ventral to the dorsal side on the right, and then back from the dorsal to the ventral side on the left. Aggregating this sequential pattern across all nine clockwise rolling events on the normalized time axis (Fig. 3B) revealed four co-active muscle groups on each side of the body that are sequentially recruited during the rolling cycle (Fig. 3B–D). We formalized the apparent groupings by creating a cladogram based on the pairwise distances between the activation curves (Fig. 3C). The co-active muscle groups consist of a ventral group of four muscles (VO15, VO16 VA26 and VO28), a lateral group of three muscles (VL12, DL4 and DO5), a dorsolateral group of three muscles (DO11, DO19 and DO20) and finally a dorsal group with one or two muscles (DL9 and/or DL2), which are sequentially activated on the right side and then on the left side of the body (Fig. 3D). Interestingly, there were possible asymmetries between the co-active groups on the left and right side (Fig. 3C). For instance, muscles DL4 and DO5 cluster with distinct sets of muscles on the right and left side. This finding should be interpreted with caution as the speed of muscle recruitment in the second half of the roll is faster than in the first half of the roll, and this leads to overlap in the timing of DL4 and D05 muscle recruitment. As a sparsely activated muscle, DL2 is not plotted in Fig. 3B–D for clarity.

We observed that the increase in fluorescence and the shortening of muscles continued until the active muscles entered the C-bend. It was not possible to resolve all of the muscles once they neared the C-bend, so we chose three muscles that are easier to identify and represent three of the co-active muscle groups: DL9, DL4 and VA26. We tracked the change in fluorescence of these muscles in segment A4, showing that the calcium signals gradually increased and reached a maximum at the C-bend, reflecting the continuous contraction of individual muscles until they reached their shortest length within the inner curve the C-bend (Fig. 3E,F).

### The sequence of muscle contractions in counterclockwise rolling is the reverse of clockwise rolling

**Fig. 4.**
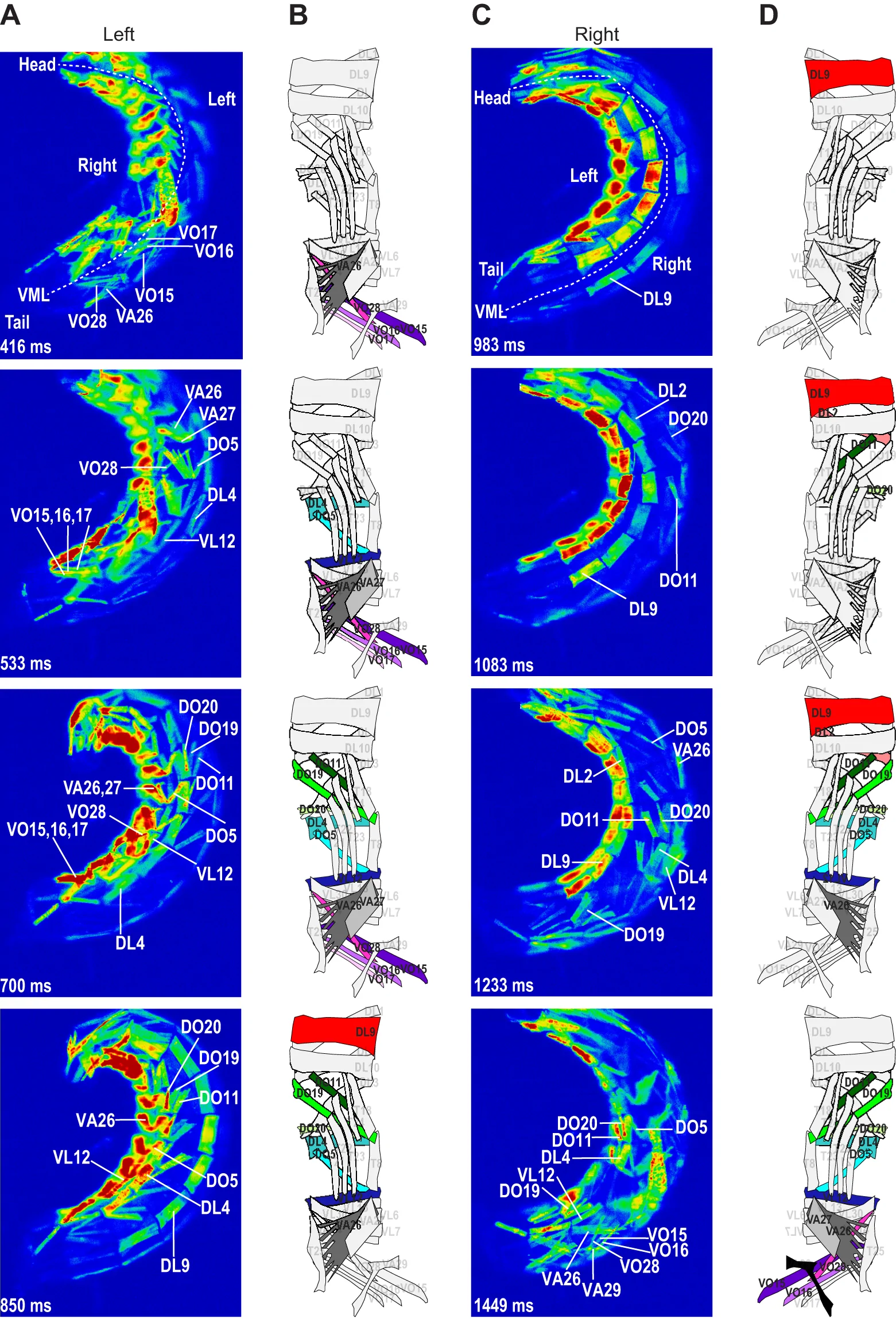
**The sequentially recruited body wall muscles during counterclockwise rolling.** (A,C) Selected frames from a recording of muscle activity of left (A) and right (C) hemisegments during counterclockwise rolling behavior. Colors from blue to red represent the intensity of the GCaMP6f signal from low to high (see Movie 2). Dashed lines indicate the ventral (VML) and dorsal (DML) midline. A subset of visibly activated homologous muscles is labeled. ‘Right’ and ‘Left’ denote the corresponding side of the larval body. (B,D) Schematic representation of the visibly activated muscles in larval abdominal left (B) and right (D) hemisegments for the frames shown in A and C. Solid colors indicate the activated muscles that are visible in the frame to the left.

**Fig. 5.**
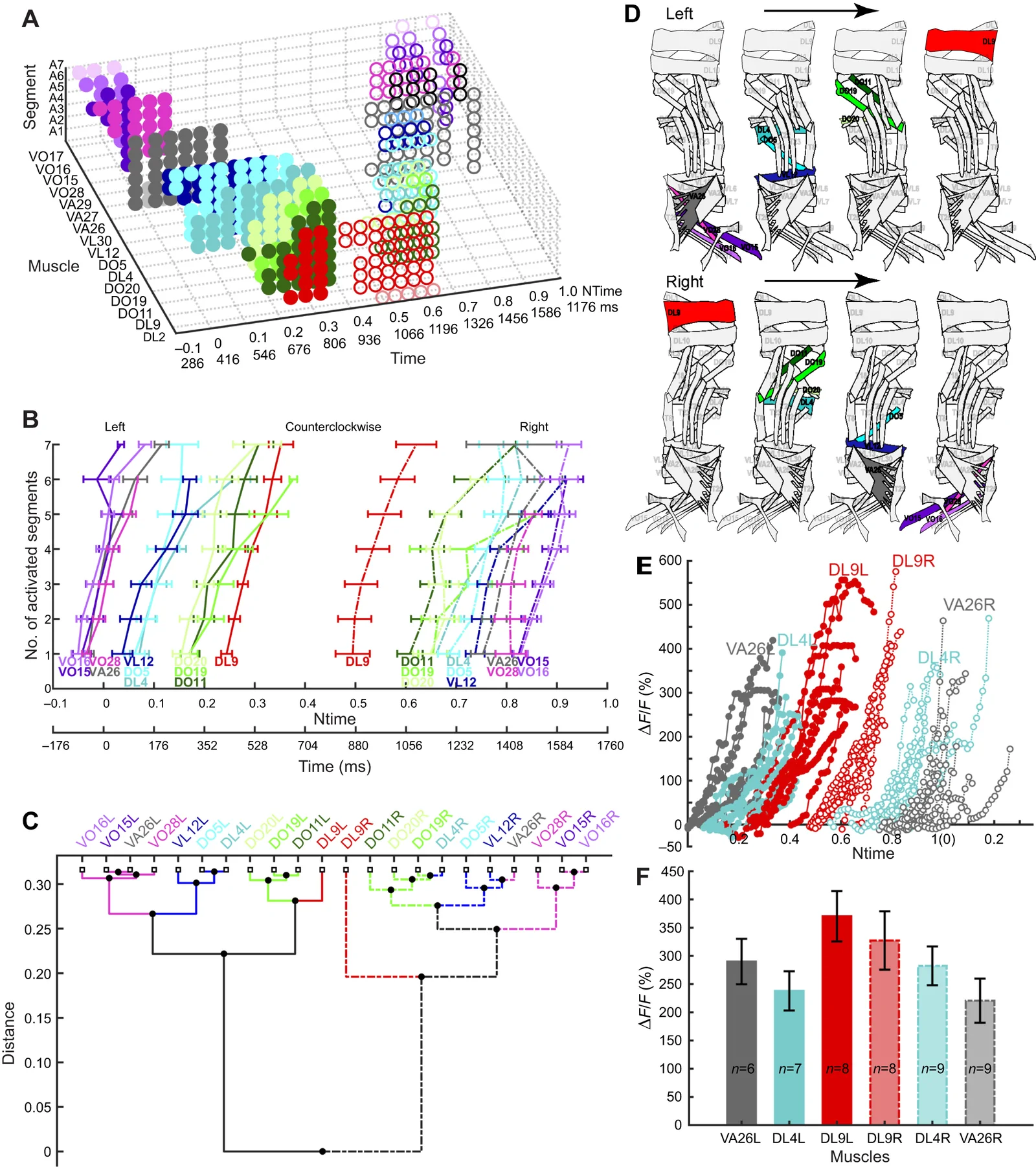
**The sequence of muscle recruitment during counterclockwise rolling.** (A) A 3D scatter plot depicting the sequence of activated muscles across the abdominal segments during counterclockwise rolling, from the left side (filled circles) to the right side (open circles). The *z*-axis shows the seven segments, the *y*-axis indicates the identity of individual muscles and the *x*-axis shows the time normalized to the rolling cycle as well as the true time from the recording (see also Movie 4). Time in ms was obtained by multiplying NTime by the average duration of a rolling cycle. (B) Plot of the average recruitment timing for the 11 abdominal body wall muscles that are activated during larval counterclockwise rolling. Error bars represent s.e.m. (*n*=10). The *x-*axis labels show the normalized rolling phase and the average minimum–maximum normalized time, respectively. (C) Cladogram depicting the relationship of recruitment timing of 11 commonly activated muscles in counterclockwise rolling behavior. Four co-activated groups of muscles are color coded. Solid branch lines denote the left side of the larva and dashed lines denote the right side of the larva. (D) Schematic drawing showing the sequential activation of co-active muscle groups, proceeding from the right to the left side of the larval body during a counterclockwise roll. (E) Time series of GCaMP6f Δ*F*/*F* in muscles VA26, DL4 and DL9 of abdominal segment A4 from nine recordings during counterclockwise rolling behavior, until the muscles disappear into the innermost curve of the C-bend. Solid lines and solid circles indicate the left hemisegment (L); dashed lines and open circles indicate the right hemisegment (R). (F) Average of the measurable highest Δ*F*/*F* across the recordings in muscles DL9, DL4 and VA26 of abdominal segment A4 during larval counterclockwise rolling. Error bars indicate the s.e.m.

During counterclockwise rolling, muscle contractions propagate towards the opposite side of the body relative to clockwise rolling. Thus, the initial flexion begins on the ventral side and recruits muscle contractions on the left side of the body, the opposite direction to that observed in clockwise rolling (Figs 4 and 5). The rolling sequence is initiated with ventral muscles VO15L, VO16L, VO28L and VA26L (Fig. 4A,B), followed by lateral muscles VL12L, DL4L and DO5L, then dorsolateral muscles DO20L, DO19L and DO11L, and eventually dorsal muscle DL9L. As with clockwise rolling, counterclockwise rolling spreads across the dorsal midline to engage dorsal muscle DL9R followed by the dorsolateral (DO11R, D19R, DO20R), lateral (DL4R, DO5R, VL12R) and eventually ventral muscle group (VO15R, VO16R, VA26R, VO28R) (Fig. 4). As with clockwise rolling, there are possible asymmetries between the co-active groups on the left and right side (Fig. 4C). Muscles DL4 and DO5 again cluster with distinct sets of muscles on the right and left side. As noted above, this switching of position in the cladogram in the second half of the roll may be a spurious result and would require further investigation for certainty. The detailed analysis of the timing of muscle activation across all segments A1–A7 (Fig. 5A), and aggregated across all 10 counterclockwise rolling events (Fig. 5B), clearly demonstrates the sequential progression of muscle activity from ventral to dorsal on the left, across the midline, and then dorsal to ventral on the right, in a mirror image of the clockwise rolling pattern. Similarly, there appear to be four co-active muscle groups in counterclockwise rolling that comprise the same sets of muscles as in clockwise rolling (Fig. 5B–D). Homologous muscles from the co-active groups co-activate across nearly all abdominal segments, as the wave of activation travels from one co-active group to the next around the circumference of the larva. Tracking the calcium signals in three representative muscles DL9, DL4 and VA26 showed a similar gradual activation which reached its maximum in the C-bend (Fig. 5E,F).

### A conceptual circuit for rolling emerges from the larval connectome

**Fig. 6.**
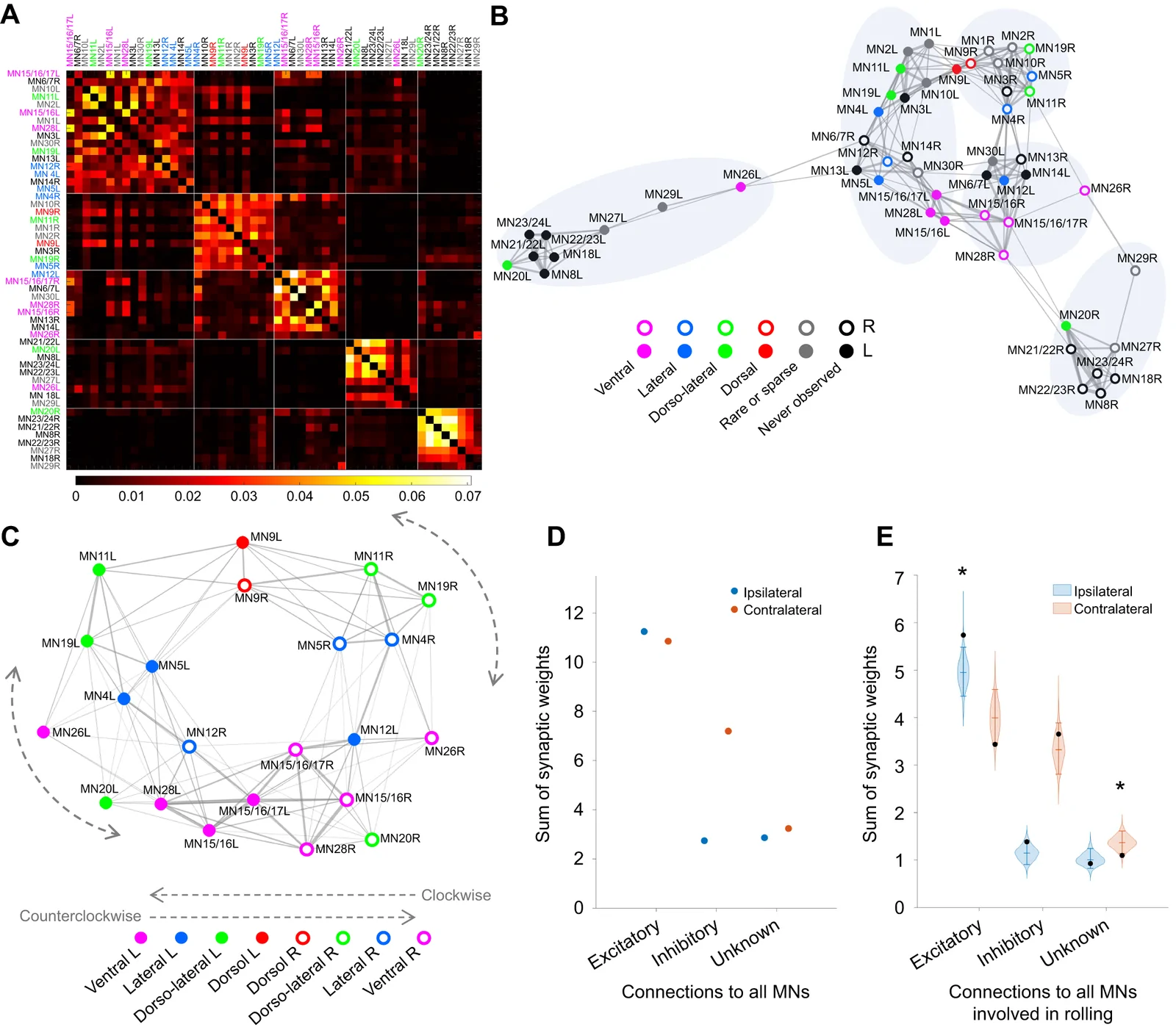
**Conceptual connectivity network for motor neurons (MNs) involved in rolling.** (A) Connectivity matrix representing the relationships between MNs based on their shared inputs from premotor neurons (PMNs). The color of each square represents the strength of the relationship between two MNs, which is the sum of synaptic input weights from shared PMNs. The MN nodes are grouped according to communities with greater connectivity, as determined by a Louvain community detection algorithm. (B) Network graph to visualize the connectivity matrix in A and reveal features of the network architecture. For visual clarity, only stronger connections are shown with a threshold of >0.015 (percentage of the total synaptic inputs to each MN). The line width of the edges represents the strength of the relationship. Node layout was determined automatically by a MATLAB algorithm. Shaded regions indicate the communities shown in A. (C) Network graph for MNs that innervate the muscles consistently involved in rolling. Only stronger connections are shown with a threshold of >0.005 (percentage of the total synaptic inputs to each MN). The line width of the edges represents the strength of the relationship. Node layout was determined automatically by a MATLAB algorithm. MN nodes are color coded in A–C as magenta for ventral co-active muscle targets, blue for lateral co-active muscle targets, green for dorso-lateral co-active muscle targets, red for dorsal muscle targets, gray for muscle targets that are rarely or sparsely active during rolling and black for muscle targets that were never active during rolling; open circles are for the right side and filled circles are for the left side. (D) The sum of synaptic weights between all PMNs and their target MNs, for excitatory, inhibitory and unknown synapses to ipsilateral or contralateral connections. (E) The sum of synaptic weights between PMNs and MNs that target muscles that are consistently activated during rolling, for excitatory, inhibitory and unknown synapses to ipsilateral or contralateral connections. Black circles show observed values for the rolling circuit, violin plots show permuted data, with error bars representing the mean and 95% confidence interval; *P<0.05, permutation test (see Materials and Methods).

We next examined the connectivity of motor neurons to reveal possible circuit structures that could support the muscle activity that controls rolling behavior. The synaptic connections of 236 PMNs and 52 MNs were previously mapped in segment A1 of an abdominal ganglion larval connectome (Zarin et al., 2019). The majority of MNs target only one muscle, except for a small number of MNs that send shared inputs to two or three related muscles. We follow the naming convention where MN numbering matches that of the muscle targets. Missing from this connectivity network is MN25, which is absent in segment A1. PMNs synapse onto MNs and onto other PMNs, whereas MNs only target muscles and do not innervate PMNs or other MNs. This means the innervation of MNs by PMNs is essentially a bipartite network. Bipartite networks consist of two sets of nodes where nodes of one set connect only to nodes of the other set. Each set of nodes can be converted into a one-mode projection that represents the relationships between the nodes based on the neighbors that they share from the other set. In order to conceptualize the pattern of inputs to the MNs, we considered the one-mode projection of MNs from the PMN-to-MN bipartite network. The resulting connectivity network represents the relationships between MNs based on their shared PMN inputs (Fig. 6A,B). It is important to note that although this graphing method allows us to visualize these MN relationships, it does not consider the complex network of PMN to PMN connections which are certain to be critical for network function.

This revealed an interesting network structure, in which the majority of MNs are interconnected in a ring-like architecture (Fig. 6A,B). This includes most of the MNs that target muscles that were consistently involved in rolling, as well as several muscles that were rarely or sparsely activated, and some muscles that were not involved. The ring-like group can be divided into three more closely connected communities, but the modularity within this group is relatively low. The two most closely connected communities lie outside the ring-like structure and consist mainly of MNs that innervate transverse muscles that were not involved in rolling (Fig. 6A,B).

We then wondered whether the connectivity for the 22 muscles that are consistently involved in rolling could support the sequential coordination of co-active muscle groups. Indeed, these MNs are also connected in a ring-like network (Fig. 6C). Furthermore, the position of MNs around the ring roughly matches the sequence of activation of co-active muscle groups during rolling (Fig. 6C). Following the color scheme used to represent co-active groups from Figs 3 and 5, a roll beginning with activation of MNs in the right ventral group (magenta open circles) that progresses to the right lateral group (blue open circles), then to the right dorsolateral (green open circles), right dorsal (red open circle), left dorsal (red solid circle), left dorsolateral (green solid circles), left lateral (blue solid circles) and finally left ventral (magenta solid circles) groups would result in clockwise body rotation. Similarly, following the activation of MNs in the opposite direction around the ring would result in counterclockwise body rotation (Fig. 6C). An interesting exception to this organization is MN12, which shares more inputs with MNs from the contralateral side. Another deviation from the pattern is MN20 and MN26, which cluster less closely with the other MNs of their co-active groups. MN20 in particular shares some strong inputs with the communities of MNs with transverse muscle targets. Although DO20 is located near these transverse muscles, they have different activity patterns; how their shared connectivity leads to such distinct activation will be interesting to investigate. Muscle VA26 is anatomically quite different from all the other muscles and likely has a unique role to play, even in relation to the other muscles in its co-active group.

How PMNs control this activation pattern remains to be determined. More PMNs are connected to the MN network than are theoretically necessary to produce the sequential activity of co-active muscle groups. We found that 102 PMNs in segment A1 synapse directly onto the 22 rolling MNs. The ring-like and interconnected relationships in the network suggest the possibility that the activity pattern for rolling is under distributed control with built-in redundancy.

We noted additional features of the PMN-to-MN network that may be important for the rolling motor circuit. PMNs that appear to be most prominently connected to the network based on the strength of their synaptic outputs tend to be inhibitory with contralateral targets. This observation is relevant because the pattern of muscle recruitment during rolling is characterized by unilateral activation of co-active muscle groups, which could be facilitated by a combination of ipsilateral excitation and contralateral inhibition. The total sum of synaptic weights for PMN inputs to the whole network of MNs is similar for ipsilateral and contralateral excitatory connections (Fig. 6D). In contrast, the sum of the synaptic weights of PMN inputs to MNs that were involved in rolling reveals an excess of ipsilateral excitation compared with contralateral excitation (Fig. 6E). The overrepresentation of ipsilateral excitation in the rolling network is significantly greater than expected by chance (permutation test, *P*=0.022), whereas the amount of contralateral excitation is not significantly different from chance (permutation test, *P*=0.08). An excess of contralateral inhibition (relative to ipsilateral inhibition) is a general feature that is found both for rolling MNs and for the whole motor network (Fig. 6D,E). The amount of inhibitory connectivity in the rolling network is not significantly different from that expected by chance (permutation test, *P*=0.142 ipsilateral, *P*=0.344 contralateral). Thus, we suggest that ipsilateral excitation may be particularly important in driving the rolling motor circuit.

## DISCUSSION

Our study contributes to investigations of larval motor programs with a detailed analysis of the activation sequence of muscles that generate the body rotation of larval rolling behavior. Building upon previous studies that quantified calcium signals in subsets of muscles during rolling (Cooney et al., 2023; Liang et al., 2025), we systematically analyzed the timing of activation of all 30 pairs of muscles in each abdominal segment A1–A7. Our results agree with previous findings that homologous muscles are activated simultaneously across body segments, and that this activation travels around the transverse circumference of the larva to generate body rotation (Cooney et al., 2023; Liang et al., 2025). Further, we focused on the onset of muscle activation as muscles contract into the C-bend, resulting in a reproducible pattern of 11 muscles that are consistently involved in rolling and are recruited sequentially as four co-active muscle groups on each side of the larva.

The direction of the roll, clockwise or counterclockwise, is determined by the direction in which the co-active muscle groups are recruited. Irrespective of the initial orientation of the C-bend, if the muscle activation progresses to the neighboring muscle group to the left of the most strongly contracted group, the larval rotation will be clockwise, whereas activation that progresses to the neighboring group to the right results in counterclockwise rotation. A mechanical model has been proposed to explain how this motor pattern generates internal torque and body rotation, even in the absence of gravity and external reaction forces from the environment (Liang et al., 2025).

The sensorimotor circuit for rolling must produce a binary and mutually exclusive outcome about the direction of the roll, but how this decision is made is unknown. With optogenetic activation of cIVda sensory neurons in the muscle imaging experiments reported here, we observed approximately equal frequencies of clockwise and counterclockwise rolls. However, we observed a strong bias in the orientation of the roll relative to the source of the optogenetic stimulus. In the vast majority of cases (19/23 recordings), we observed that the muscle contractions strengthened on the side of the body as the larvae rotated towards the surface of the coverslip (the side closer to the light source). Only four cases (17% of observed rolls) rotated in the other direction, with strengthening contractions on the side of the body further away from the light source and with relaxation of the contractions occurring on the side closer to the coverslip. This finding is consistent with our prior studies showing that larvae roll towards the side of the body which is receiving nociceptive input (Hwang et al., 2007). We hypothesize that because the light intensity is stronger on the side of the larva that is closer to the coverslip, the nociceptors on that side are activated more strongly by the optogenetic stimulus, and this biases the rotation of movement towards the surface of the coverslip. This directional bias allowed us to characterize muscles as they contracted and entered the C-bend, but we were unable to systematically analyze muscles as they exited the C-bend during the relaxation phase because of the rare occurrence of rolls with this orientation. Interestingly, Cooney et al. (2023) triggered rolls with optogenetic activation of the Goro command-like interneurons, which resulted in rolls with orientations both towards the direction of the light source and away from the light source. This raises the possibility that triggering the behavior from further downstream in the sensorimotor circuit means it lacks the somatotopic information that comes from local activation of the nociceptive sensory neurons to produce directional rolling.

Through our careful frame-by-frame and muscle-by-muscle analysis across a total of 19 roll events, we found variability in the muscle activation pattern with eight muscles that were either rarely or sparsely activated across segments. This variability could be due to individual differences in neuromuscular wiring, stochastic muscle activation unrelated to the rolling motor program, or flexibility in the motor program perhaps modulated by sensory feedback. Multimodal sensory information is integrated as early as first-order interneurons (e.g. Basins; Ohyama et al., 2015) in the sensorimotor rolling circuit, but it will also be important to investigate how ongoing sensory and proprioceptive feedback influence the progression and completion of rolls. In addition, flexibility, modulation and compensation are important factors to keep in mind when performing silencing and ablation experiments to identify the contribution of individual muscles, MNs or PMNs.

There remained a set of 11 muscles in which we observed no activity in any of the roll events analyzed. Therefore, only a subset of muscles is involved in rolling behavior when it is triggered by cIVda neurons (in contrast to crawling, which recruits all abdominal muscles (Zarin et al., 2019). Of particular note is the absence of activity observed in transverse muscles T21, T22 and T23 in our recordings. Previous work has suggested that the transverse muscles contribute to rolling because of innervation from the mCSI nociception pathway through SNa MNs (Yoshino et al., 2017). Silencing MN input to the transverse muscles, or ablating transverse muscles, results in a partial defect in rolling (Cooney et al., 2023; Liang et al., 2025; Yoshino et al., 2017). Quantification of muscle activity in the study by Cooney et al. (2023) as the muscles relax and emerge from the C-bend shows increased fluorescence in a subset of transverse muscles as they near the stretched side of the C-bend (opposite to the phase of the majority of other muscles). Although unlikely, it is possible that the phase of the roll in which transverse muscles contract was not visible in our recording setup. It has been proposed that while transverse muscles are not required for creating the sequential bending and rotation of the larval body, they may contribute to rolling by maintaining adequate internal pressure and rigidity (Cooney et al., 2023; Liang et al., 2025). Our analysis of the motor connectivity network shows that the MNs innervating lateral transverse muscles form closely connected communities that are separate from the ring-like relationships of the other MNs. This further supports the idea that the transverse muscles have a distinct functional role to play and is consistent with the prior indication that they may be activated out of phase with the muscles that are recruited into the C-bend.

The circumferential wave of muscle activity in rolling behavior stands in stark contrast to the longitudinal peristaltic waves of muscle activity in crawling. During larval crawling, co-active muscle groups activate sequentially within a segment, and this bilaterally symmetric pattern propagates from one segment to the next, from tail to head in forward crawling, and from head to tail in backward crawling (Zarin et al., 2019). Further, our findings indicate that the co-active muscle groups for rolling are distinct from those for crawling. The motor program for crawling therefore requires circuitry not only to generate a specific muscle activation pattern but also for intersegmental propagation. Meanwhile, the motor program for rolling is primarily based on muscle identity and requires simultaneous recruitment across all segments and dorsoventral propagation. We expect to see these differences reflected in the circuit architecture that controls these movements. It is likely that homologous sets of neurons across segments must be co-activated to recruit the homologous sets of muscles that are involved in rolling. While crawling circuits are expected to have bilateral excitation of co-active muscle groups, with simultaneous inhibition of neighboring segments, excitatory connections in rolling are expected to be unilateral and intersegmental, with simultaneous inhibition of radially opposed muscle groups. Indeed, in our conceptual rolling network of MNs and PMNs, we observed a relatively greater proportion of ipsilateral excitatory connections as well as contralateral inhibitory connections. At this time, the connectome is fully mapped only in segment A1 of the larval ventral nerve cord. It will be interesting to investigate, in follow-up network analyses, how premotor interneurons connect between and across segments. While one approach to understanding the function of the rolling and crawling circuits is to pick out individual interneurons or small circuit motifs from the larger network that have predicted circuit characteristics, this is likely to overlook weaker connections and unexpected patterns of connectivity. Instead, we sought to gain an overview of the general network organization by visualizing the shared pattern of PMN inputs to MNs that innervate the muscles involved in rolling. The network revealed an interesting ring-like structure that could support the sequential muscle activity for rolling. Whether this network can be understood as a collection of microcircuits, or whether function is distributed across the entire network, remains to be seen. A necessary next step is to identify which premotor neurons and interneurons are actually involved in rolling, through reconstruction of the whole sensorimotor nociception circuit, as well as functional validation. Advancing our understanding of the neuromuscular control of different larval movements will help us uncover general principles and features of neural circuits that control and modulate motor output in the context of a relatively small nervous system and simple body plan.

## Supplementary Material

10.1242/jexbio.252063_sup1 Supplementary information
