# Supplementary material for: The motor pattern of rolling escape locomotion in Drosophila larvae
Source: J Exp Biol. 2026 Sep 4;229(17):jeb252063. doi: 10.1242/jeb.252063 (PMC13615696; doi:10.1242/jeb.252063)
Supplement: Supplementary file 1 [file jexbio-229-252063-s1.pdf]

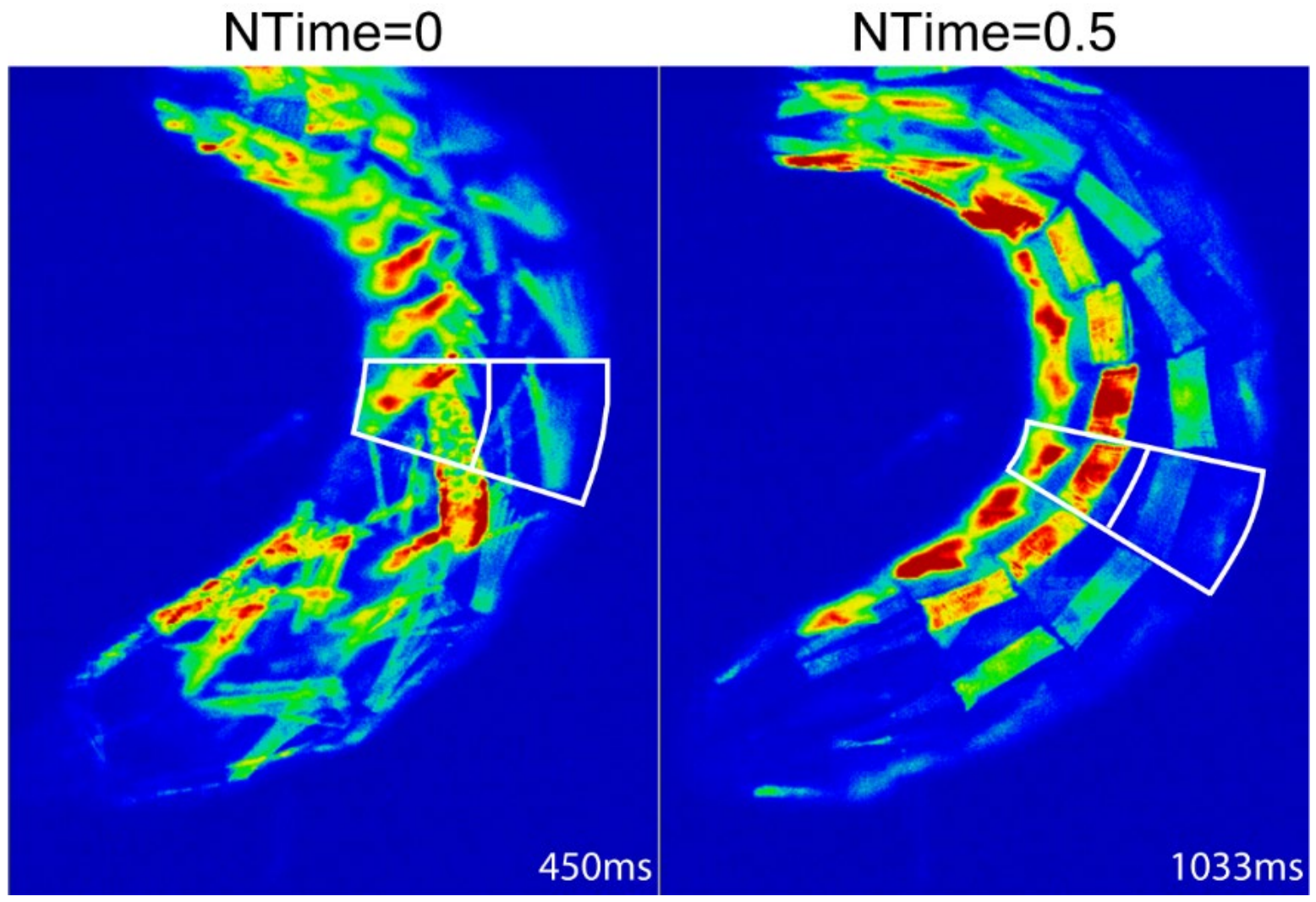

Fig S1. Larval rolling phase

NTime=(TimeR-TimeV)/ΔTime, where NTime is normalized time and TimeR is a single timestamp from a frame in the recording. The duration of a rolling cycle (ΔTime) was calculated as the time interval between two recording time points (at the start and end of a roll) when the ventral midline in segment A4 was centered in the image plane. The timestamp at the beginning of the rolling cycle was defined as TimeV. At NTime=0 the ventral midline is centered, at NTime=0.5, the dorsal midline of the larva is centered.

Journal of Experimental Biology • Supplementary information

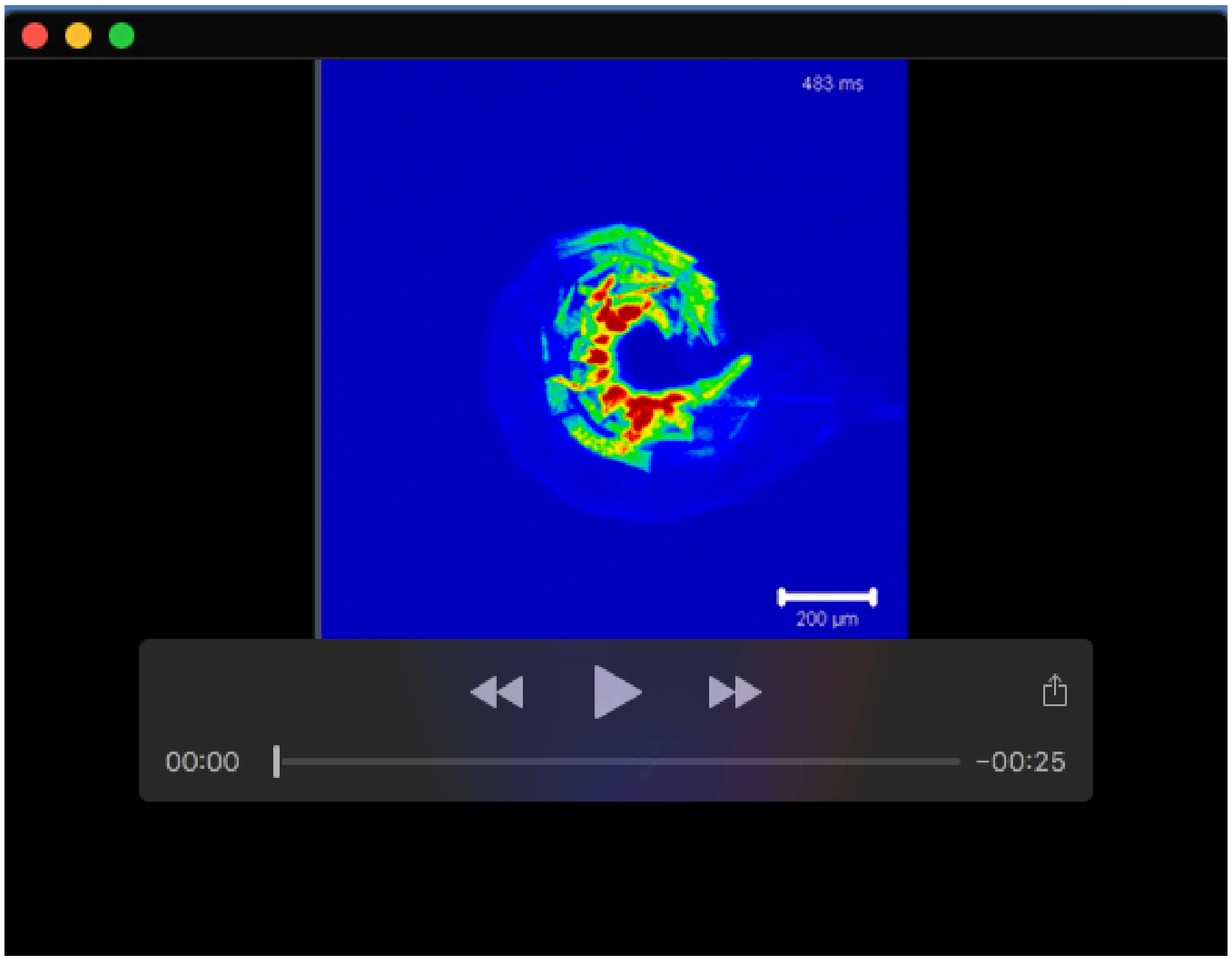

Movie 1. Recording of larval clockwise rolling. Related to **Fig 2**. Pseudocolor ranging from blue to red denotes the GCaMP6f signal from low to high. Video was recorded at 60fps, the playback rate was set as 10fps.

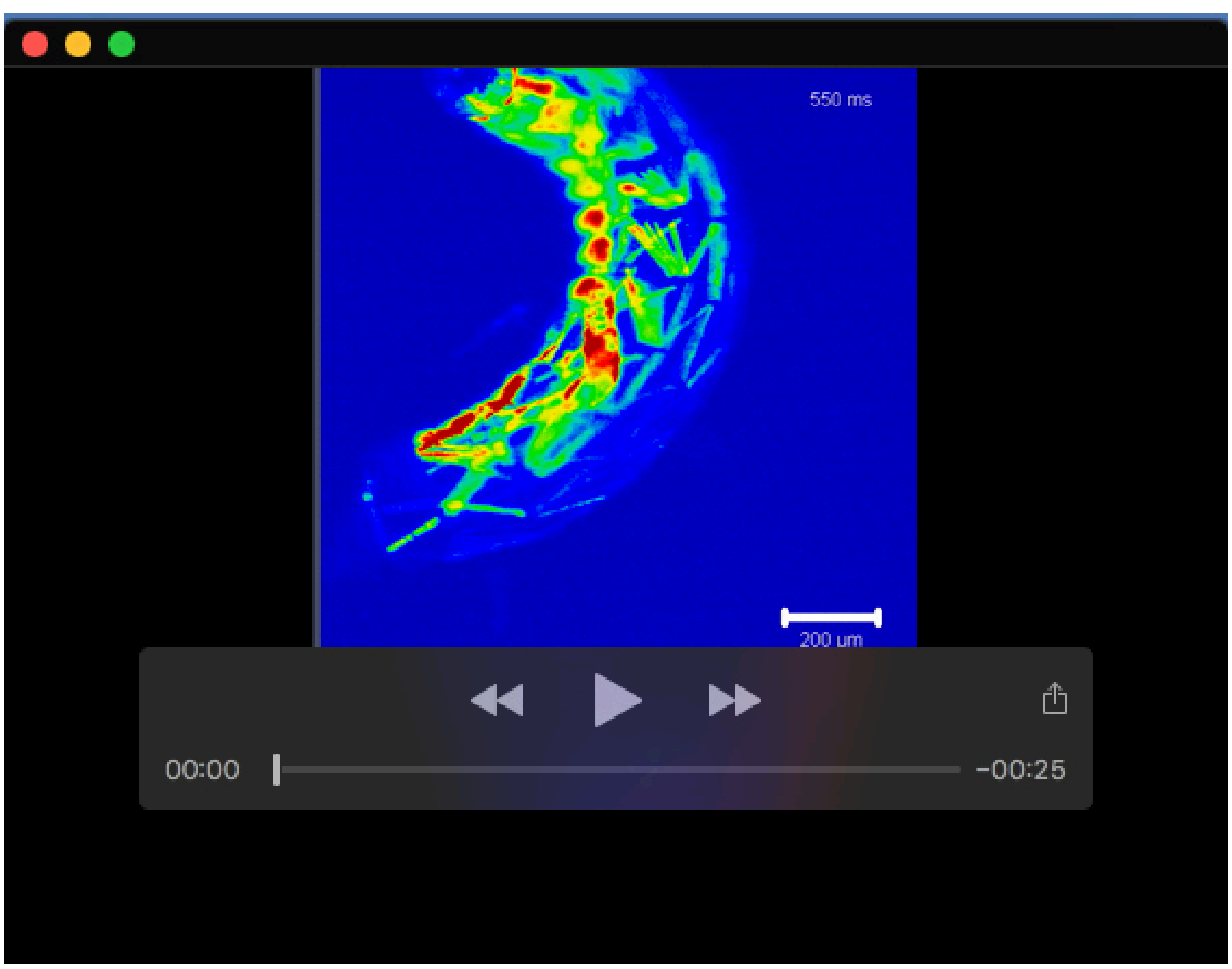

Movie 2. Recording of larval counterclockwise rolling. Related to **Fig 4**. Pseudocolor ranging from blue to red denotes the GCaMP6f signal from low to high. Video was recorded at 60fps, the playback rate was set as 10fps.

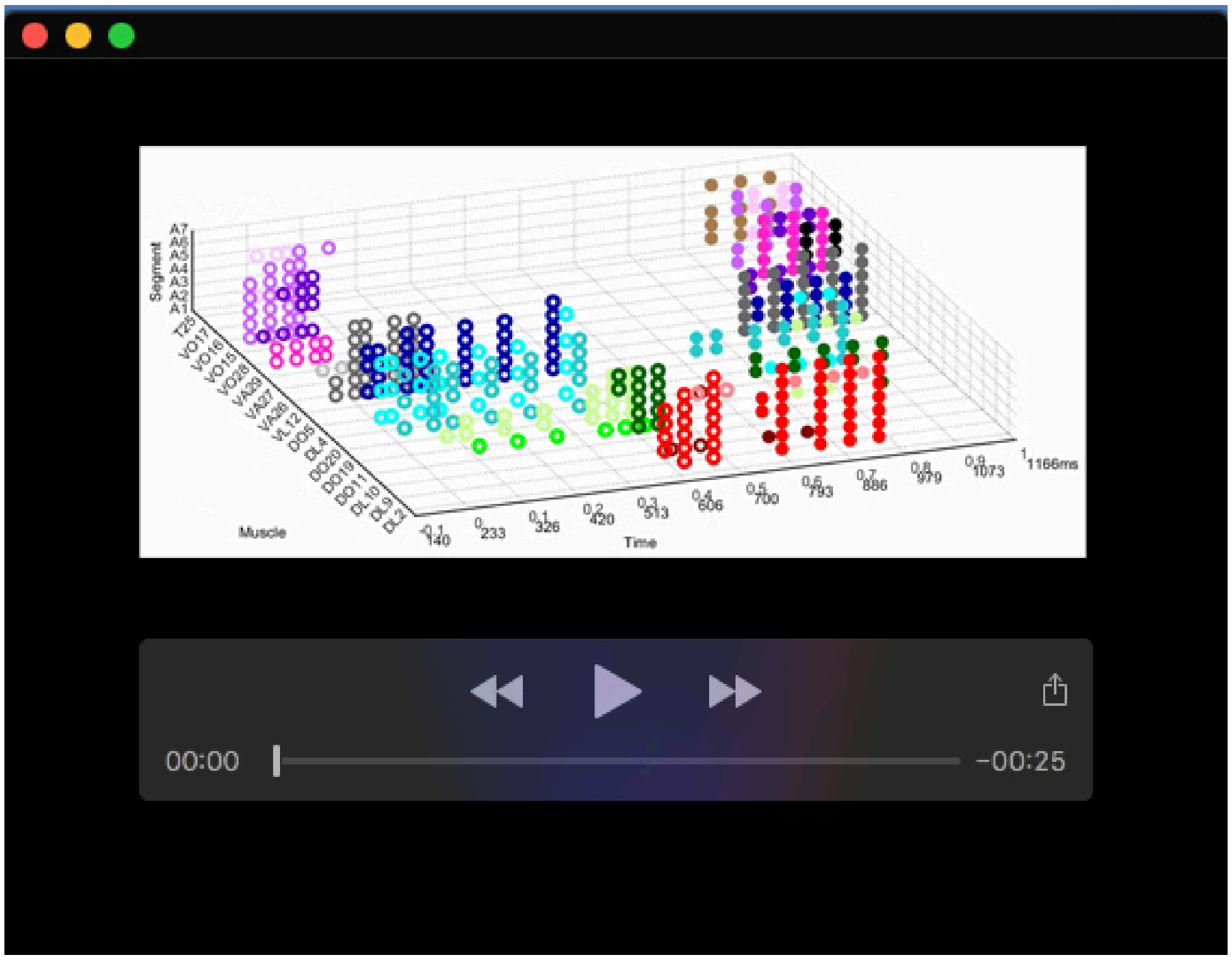

Movie 3. Video of rotating 3D plot of Figure 3A. Related to **Fig 3.**

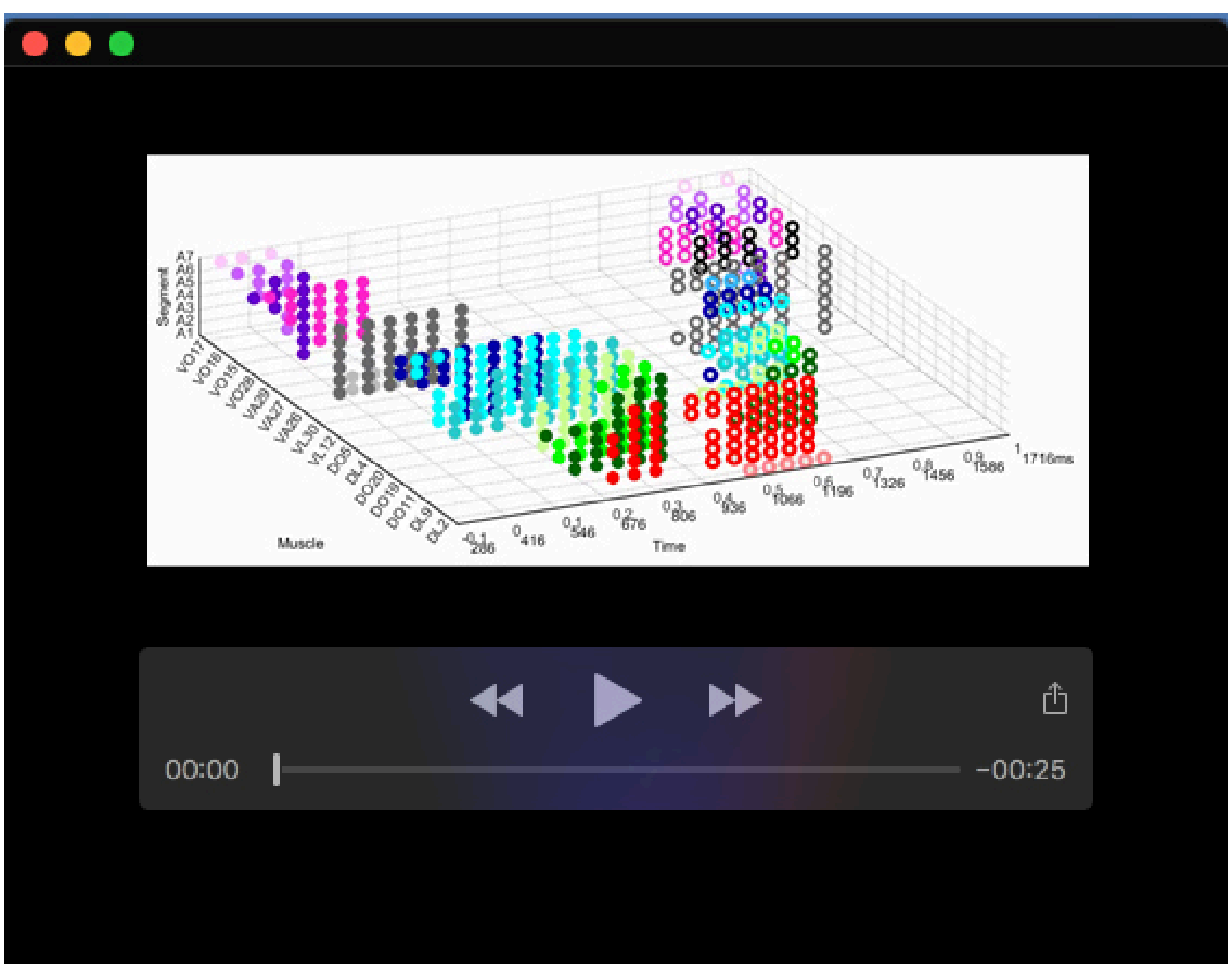

Movie 4. Video of rotating 3D plot of Figure 5A. Related to **Fig 5.**
